# ﻿Taxonomic corrections, new records, and life history information on the jumping tree bugs (Hemiptera, Miridae, Isometopinae) of Korea

**DOI:** 10.3897/zookeys.1251.156931

**Published:** 2025-09-05

**Authors:** Wonwoong Kim, Minsuk Oh, Geonho Cho

**Affiliations:** 1 Department of Ecology and Evolutionary Biology, University of Michigan, Ann Arbor, MI 48109, USA; 2 Insect Biosystematics Laboratory, Department of Agricultural Biotechnology, Seoul National University, Seoul 151-921, Republic of Korea; 3 Research Institute of Agriculture and Life Sciences, Seoul National University, Seoul 151-921, Republic of Korea; 4 Department of Forest Resources, Sunchon National University, Suncheon 57922, Republic of Korea

**Keywords:** Biodiversity, distributions, East Asia, plant bugs, synonymy

## Abstract

The identities of two species of Korean Isometopinae (Hemiptera: Miridae) are clarified based on examination of their type material. The following new synonyms are proposed: *Isometopus amurensis* Kerzhner, 1988 = *Isometopus jejuensis* Kim & Jung, 2016, **syn. nov.** and *Myiomma ussuriense* Ostapenko, 2001 = *Myiomma koreanum* Oh & Lee, 2025, **syn. nov.***Isometopus citri* Ren, 1987 is reported from Korea for the first time. Life histories of *I. amurensis* Kerzhner, *I. japonicus* Hasegawa, 1946, and *I. rugiceps* Kerzhner, 1988 are documented based on Korean individuals.

## ﻿Introduction

A number of taxonomic and faunistic studies on jumping tree bugs (Hemiptera: Miridae: Isometopinae) of Korea have been conducted, despite the relatively recent discovery of this group from this region (Jung et al. 2015; Kim and Jung 2016; Ahn et al. 2018; Oh and Lee 2019; Oh et al. 2025). To date, four *Isometopus* Fieber, 1860 and one *Myiomma* Puton, 1872 species have been recognized in Korea. Examinations of photographs of the type material housed in the Zoological Institute, Russian Academy of Sciences, St. Petersburg, Russia, revealed that taxonomic corrections are needed for the recently described species from Korea. In addition, we herein update ecological and distributional data for the Korean jumping tree bug species.

## ﻿Materials and methods

### ﻿Specimen collection

Field surveys were mainly conducted from June 2023 to July 2024. Most specimens were hand-collected by visual inspections of the tree bark with an aspirator. Final-instar nymphs were collected and reared with the plant bark of the respective collection site, mainly following the methodology of Tsunoda et al. (2020) by providing fermented milk beverage and brine shrimp eggs, supplemented with dead insects such as bark lice. Additional specimens were collected on light traps.

### ﻿Morphological observation

Live specimens were photographed using Nikon D5600 or Nikon Z6ii equipped with Nikon AF-S VR Micro Nikkor ED 105 mm f/2.8G lens. The morphological terminology follows Yasunaga et al. (2017). For the type labels, the label data are cited verbatim, where individual lines are divided by ‘\’. Images of the type specimens of *Isometopus amurensis* Kerzhner, 1988 and *Myiomma ussuriense* Ostapenko, 2001 from Zoological Institute, Saint Petersburg, Russia were either retrieved from Konstantinov and Neimorovets (2025) or directly from the institution.

### ﻿Ecological data

Data on the distribution and collection information for East Asian *Isometopus* species were compiled from Kerzhner (1988a, 1988b), Yasunaga (2001, 2005), Qi (2005), Kim and Jung (2016), Yasunaga et al. (2017), Urayama et al. (2019), and Tsunoda et al. (2020). The scientific names of the vascular plants were updated following Kew Plants of the World Online database (POWO 2025).

### ﻿Molecular data and phylogenetic analysis

Genomic DNA was extracted using a DNeasy Blood and Tissue kit (Qiagen, Germany), following the manufacturer’s protocol. Both the nymphs and the adults were immersed in the lysis buffer with proteinase K solution overnight at 56 °C. After incubation, the specimens were removed, cleaned, and stored in 99% ethanol at SCNU for future reference. The mitochondrial cytochrome c oxidase subunit I (COI) was amplified using the polymerase chain reaction (PCR) with the primers LCO1490F (5-GGTCAACAAATCATAAAGATATTGG-3) and HCO2198R (5-GGTCAACAAATCATAAAGATATTGG-3) following the thermal cycling condition for Miridae following Oh et al. (2023) of initial denaturation at 95 °C for 2 min, followed by 38 cycles of denaturation at 95 °C for 30 s, annelation at 45–55 °C for 30 s, extension at 72 °C for 1 min, and final extension at 72 °C for 10 min. The amplicons were purified and sequenced in Bionics Co. (Seoul, Korea). Consensus sequences were generated in Geneious Prime software, and multiple sequence alignment was performed in MAFFT online (Katoh et al. 2017). Final sequence assessment was performed in MEGA X (Kumar et al. 2018). A total of 20 sequences were used, of which 14 were newly sequenced and six were obtained from GenBank (Table 1). Phylogenetic analysis was conducted in raxmlGUI 2.0 (Edler et al. 2020) using ML+rapid bootstrap option with 1000 reps. The resulting tree was visualized in FigTree v. 1.4.4 (Rambaut 2018). The genetic distances of the COI sequences were calculated using *p*-distance option in MEGA X.

**Table 1.** Details of selected COI sequences used for the phylogenetic analysis.

Species	GenBank accession number	Locality	Reference
*Sophianus lamellatus* Ren & Yang, 1988	MZ408091.1	Cambodia	Oh et al. (2023)
*Myiomma ussuriense* Ostapenko, 2001	PX056004	Suwon, Korea	This work
*Isometopus amurensis* Kerzhner, 1988	KY367033.1	Ansan, Korea	Kim and Jung (2018)
*Isometopus amurensis* Kerzhner, 1988	MZ408037.1	Anyang, Korea	Oh et al. (2023)
*Isometopus amurensis* Kerzhner, 1988	PX055995	Incheon, Korea	This work
*Isometopus amurensis* Kerzhner, 1988	PX055991	Anyang, Korea	This work
*Isometopus amurensis* Kerzhner, 1988	PX055994	Suwon, Korea	This work
*Isometopus amurensis* Kerzhner, 1988	PX055993	Ilsan, Goyang, Korea	This work
*Isometopus amurensis* Kerzhner, 1988	PX055992	Gwacheon, Korea	This work
*Isometopus jejuensis* Kim & Jung, 2016	KY367037.1	Jeju, Korea	Kim and Jung (2018)
*Isometopus japonicus* Hasegawa, 1946	KY367035.1	Jangseong, Korea	Kim and Jung (2018)
*Isometopus japonicus* Hasegawa, 1946	PX055997	Pocheon, Korea	This work
*Isometopus japonicus* Hasegawa, 1946	PX055998	Seocho, Seoul, Korea	This work
*Isometopus japonicus* Hasegawa, 1946	PX055999	Gwacheon, Korea	This work
*Isometopus japonicus* Hasegawa, 1946	PX056000	Ilsan, Goyang, Korea	This work
*Isometopus japonicus* Hasegawa, 1946	PX055996	Siheung, Korea	This work
*Isometopus rugiceps* Kerzhner, 1988	PX056001	Anyang, Korea	This work
*Isometopus rugiceps* Kerzhner, 1988	PX056002	Gwacheon, Korea	This work
*Isometopus rugiceps* Kerzhner, 1988	PX056003	Sancheong, Korea	This work
*Isometopus rugiceps* Kerzhner, 1988	MZ408038.1	Inje, Korea	Oh et al. (2023)

### ﻿Depositories of specimens

The specimens examined or mentioned in this study are deposited in the following institutions:

**CNU** Laboratory of Systematic Entomology, Chungnam National University, Daejeon, Korea;

**NIAES**National Institute for Agro-Environmental Sciences, Tsukuba, Japan;

**NIBR**National Institute of Biological Resources, Incheon, Korea;

**NKUM**Institute of Entomology, Nankai University, Tianjin, China;

**SCNU** Department of Forest Resources, Sunchon National University, Suncheon, Korea;

**SNU**Insect Biosystematics Laboratory, Seoul National University, Seoul, Korea;

**UMMZ**Museum of Zoology, University of Michigan, Ann Arbor, USA;

**ZISP**Zoological Institute, Russian Academy of Sciences, St. Petersburg, Russia.

## ﻿Results

### ﻿Taxonomy

**Family Miridae Hahn, 1833**

**Subfamily Isometopinae Fieber, 1860**

**Tribe Isometopini Fieber, 1860**

#### ﻿Genus *Isometopus* Fieber, 1860

##### ﻿ Isometopus amurensis

Taxon classificationAnimaliaHemipteraMiridae

Kerzhner, 1988

69AE9682-9336-5CC5-891A-D55682B85C24

**Figure 1.**
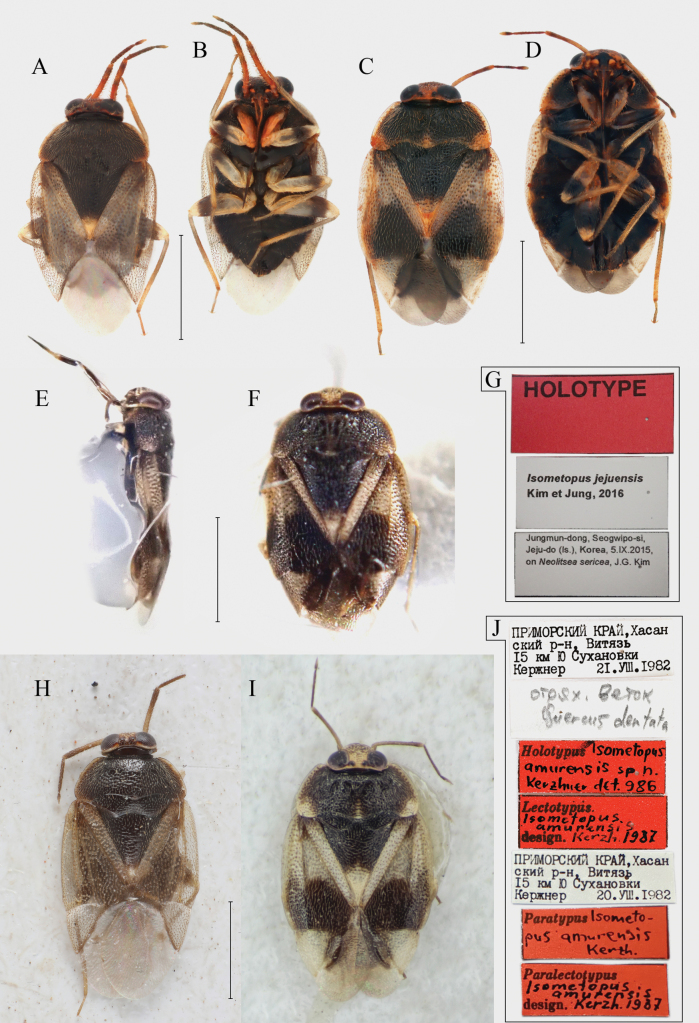
A–D. *Isometopus amurensis* Kerzhner, 1988 E–G. *I. jejuensis* Kim & Jung, 2016. A. Non-type male from Gwacheon, Korea, dorsal view; B. Same, ventral view; C. Non-type female, same locality, dorsal view; D. Same, ventral view; E. Holotype female, lateral view; F. Same, dorsal view; G. Label; H. Lectotype male of *I. amurensis*, dorsal view; I. Paralectotype female of *I. amurensis*, from Vityaz’, Primorsky Krai, Russia, dorsal view; J. Label (lectotype, paralectotype). Scale bars: 1.0 mm. © ZISP (H–J).

**Figure 2.**
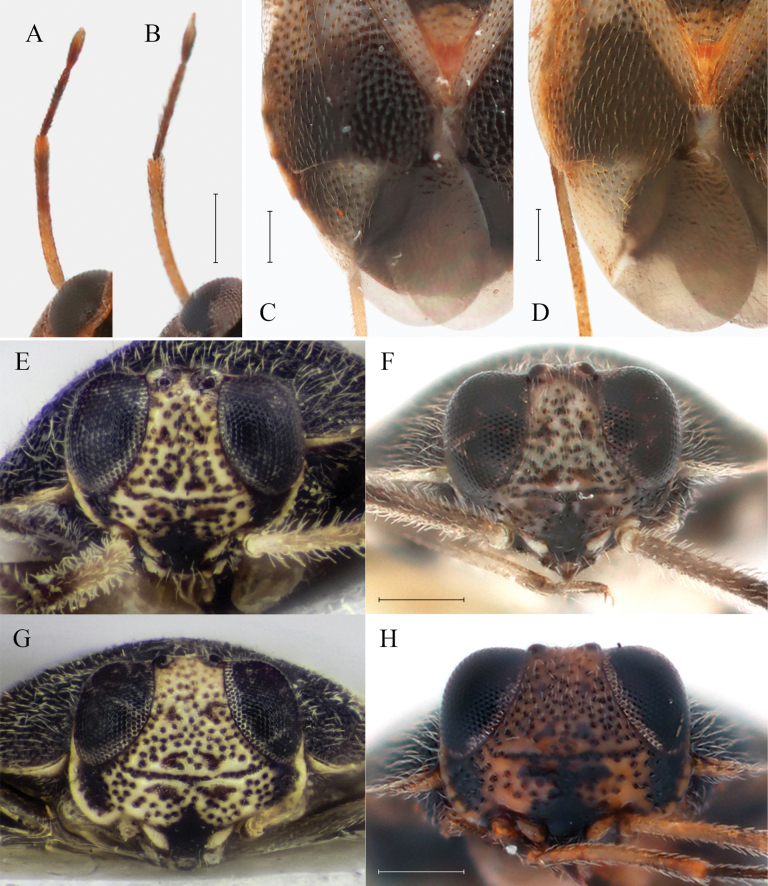
*Isometopus amurensis* Kerzhner, 1988. A–D, F, H. Non-type specimens from Korea. E, G: type specimens from Primorsky Krai, Russia. A, B. Antennae, female; C, D. Hemelytral coloration, female; E. Head in frontal view, lectotype male; F. Head in frontal view, male; H. Same, female; G. Same, paralectotype female. Scale bars: 0.2 mm. © ZISP (E, G).

**Figure 3.**
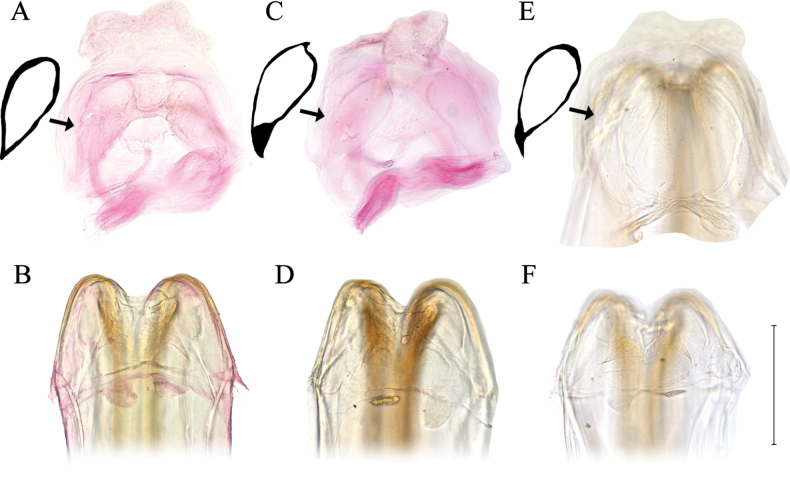
Female genital structure of *Isometopus amurensis* Kerzhner, 1988. A, C, E. Sclerotized ring; B, D, F. Posterior wall. Collection localities: A–D. Gwacheon-si, Korea (37.425414, 126.987958); E, F. Suwon-si, Korea (37.300349, 127.030048). Scale bar: 0.2 mm.

Figs 1A–J, 2A–H, 3A–F, 6A–G

Isometopus amurensis Kerzhner, 1988a: 789. Lectotype (as holotype, see Kerzhner 1988b: 4): ♂, Russia: Vityaz’, 15 km south of Sukhanovka; ZISP.
Isometopus jejuensis Kim & Jung, 2016: 138. Holotype: ♀, Korea: Jungmun-dong, Jeju Island; CNU. syn. nov.
Isometopus amurensis : Kerzhner 1988b: 4 (redescription); Kerzhner and Josifov 1999: 3 (catalogue, distribution); Schuh 2002–2013 (web catalogue); Jung et al. 2015: 598 (distribution, Korea); Ahn et al. 2018: 381 (diagnosis, photo); KSAE and ESK 2021: 155 (list); Taszakowski et al. 2023: 185 (catalogue).
Isometopus jejuensis : KSAE and ESK 2021: 155 (list); Taszakowski et al. 2023: 186 (catalogue).

###### Type material examined.

***Isometopus amurensis*. *Lectotype***: ♂, “[PRIMORSKY KRAI, Khasan \ District, Vityaz’ \ 15 km S of Sukhanovka \ Kerzhner 21.viii.1982]” [printed], back of the label: “[shaking off branches of Quercus dentata]” [handwritten] “*Holotypus*Isometopus \ amurensis sp. n. \ Kerzhner det. 986” [printed + handwritten] “*Lectotypus* \ Isometopus \ amurensis \ design. Kerzh. 1987” [printed + handwritten] also with ZISP catalogue number: INS_HEM_0000123. ***Paralectotype***: ♀, “[PRIMORSKY KRAI, Khasan \ District, Vityaz’ \ 15 km S of Sukhanovka \ Kerzhner 20.viii.1982]” [printed], “*Paratypus* Isometo- \ pus amurensis \ Kerzh.” [printed + handwritten] “*Paralectotypus* \ Isometopus \ amurensis \ design. Kerzh. 1987” [printed + handwritten], both deposited in ZISP, photographs examined. ***Isometopus jejuensis*. *Holotype***: ♀, “Jungmun-dong, Seogwipo-si, \ Jeju-do (Is.), Korea, 5.IX.2015, \ on *Neolitsea sericea*, J.G. Kim” [printed] “Isometopus jejuensis \ Kim et Jung, 2016” [printed] “HOLOTYPE” [printed], deposited in CNU.

###### Additional material examined.

South Korea – **Gyeonggi-do.** • 1 ♂; 12, Byeoryang-ro, Gwacheon-si; 37°25.26'N, 126°59.42'E; on light; 15.viii.2019; W. Kim leg. (SNU) • 1 ♀; Seoul National University Gwanak Arboretum, Anyang-si; 05.viii.2015; M. Oh leg. (SNU) • 1 ♂, 2 nymphs (5^th^); Jungang-dong, Gwacheon-si; 37°25.52'N, 126°59.27'E; 17.vi.2023; W. Kim leg. (UMMZ); • 4 ♂♂, 4 ♀♀, 2 nymphs (5^th^); same locality; 21.vi.2023; W. Kim leg. (SNU) • 1 nymph; Manan-gu, Anyang-si; 37°25.96'N, 126°54.92'E; 21.vi.2024; W. Kim leg. (UMMZ) • 2 nymphs; Ilsan lake park, Goyang-si; 37°39.17'N, 126°45.85'E; 20.vi.2024; W. Kim leg. (SCNU) • 5 nymphs (four 5^th^); Gwanggyo Park, Jangan-gu, Suwon-si; 37°18.02'N, 127°01.80'E; 22.vi.2024; W. Kim and M. Choi leg. (SCNU) • 1 ♂, 2 ♀♀, 4 nymphs (two 5^th^); same locality; 26.vi.2024; W. Kim and M. Oh leg. (SNU) • 5 ♂♂, 4 ♀♀, 1 nymph; Jungang-dong, Gwacheon-si; 37°25.52'N, 126°59.27'E; 26.vi.2024; W. Kim leg. (SNU, SCNU) • 1 ♂, 2 ♀♀, 2 nymph (one 5^th^); same locality and collector; 28.vi.2024 (UMMZ). – **Incheon.** • 1 ♂, 2 nymphs; Sorae Wetlands Ecology Park, Nonhyeon-dong, Namdong-gu; 37°24.55'N, 126°44.72'E; 14.vi.2023; W. Kim leg. (SCNU). – **Jeollanam-do.** • 1 ♀, 2 nymphs (5^th^); Yongdang-dong, Suncheon-si; 34°58.30'N, 127°29.29'E; 7.vii.2024; W. Kim leg. (SCNU). – **Jeju-do.** • 2 nymphs; Hoecheon-dong, Jeju-si; 33°26.96'N, 126°38.36'E; 5.vii.2025; W. Kim leg. (UMMZ).

###### Diagnosis.

Sexually dimorphic (Figs 1, 2, 6). Easily distinguished from congeners in East Asia by the light brown frons and distinct punctation pattern (Fig. 2E–H); hyaline hemelytra, and apically white scutellum in males; yellowish-white hemelytra with a dark band on the posterior half of corium in females; nymphs as in Fig. 6E–G. As suggested by Kerzhner (1988b), this species is externally similar to *I. tianjinus* Hsiao, 1964, but *I. amurensis* can be distinguished by the tip of the left paramere not swollen in male, and the black scutellum except for the white apical 1/3–1/4 in females (Hsiao 1964; Kerzhner 1988b; Liu et al. 2022). However, since the known descriptions and figures for *I. tianjinus* are brief, future works are needed to clearly elucidate the diagnostic characters between *I. tianjinus* and *I. amurensis*.

###### Distribution.

Russia (Far East: Amur Oblast, Primorsky Krai) (Kerzhner 1988b), South Korea (Gyeonggi-do, Gangwon-do, Jeollabuk-do, Jeollanam-do, Jeju-do) (Jung et al. 2015; Kim and Jung 2016; Ahn et al. 2018).

### ﻿Species status of *Isometopus jejuensis* Kim & Jung, 2016

*Isometopus amurensis* Kerzhner, 1988 was described based on a male holotype and an additional male and three female paratypes from the Russian Far East (Amur Oblast, Primorsky Krai). The original description states that *I. amurensis* resembles *I. tianjinus* Hsiao, 1964 from northern China (Liu et al. 2022) and *I. kaznakovi* Kiritshenko, 1939 from the Caucasus (Kerzhner and Josifov 1999).

*Isometopus jejuensis* Kim & Jung, 2016 was described based on a female holotype and an additional 11 female paratypes collected from a single locality in Jungmun-dong, Jeju Island, South Korea. The authors discussed that their new species resembles *I. amurensis* but can be distinguished by 1) the dark brown antennal segment II, 2) the unique shape of female sclerotized rings, and 3) different host plants. They also compared the new species with *I. nigrosignatus* Ren, 1987 (from Yunnan province in China and Taiwan, cf. Lin and Yang 2004; Liu et al. 2022) and *I. mirificus* Mulsant & Rey, 1879 (from various parts of Europe, cf. Wagner and Weber 1964; Kerzhner and Josifov 1999) and noted distinctions in the color pattern of dorsum. However, Kim and Jung (2016)’s mention of *I. mirificus* from Ishigaki Island, Japan, is likely an oversight of literature: the species from Ishigaki was tentatively reported as *I. mahal* (Distant, 1911) by Yasunaga and Hayashi (2002) and later recognized as a new species, *I. ishigaki* Yasunaga, 2012, which is known only from the Yaeyama Island group of the Ryukyus that inhabits the bark of *Fraxinus griffithii* C. B. Clarke (Oleaceae) (Yasunaga 2005; Yasunaga et al. 2012, 2017). We also note that *I. mahal* was synonymized with *I. mirificus* by Carvalho (1951), but later resurrected by Akingbohugbe (1996). Its current distribution is now restricted to Karnataka and West Bengal states in India (Yeshwanth et al. 2021). Therefore, the accurate name for the species in Ishigaki Island is *I. ishigaki*, instead of *I. mirificus*.

Both *I. nigrosignatus* and *I. ishigaki* bear superficial similarity with *I. jejuensis* in terms of the ‘black and white’ dorsal coloration; they clearly differ from *I. jejuensis* by the general body shape and color pattern of the frons. Rather, *I. nigrosignatus* and *I. ishigaki* are known to be related to *I. mahal* from India (Yasunaga 2005; Yeshwanth et al. 2021), and are also similar to *I. hainanus* Hsiao, 1964 from Hainan Island, China (see Liu et al. 2022: 432–433, pl. xvii).

Many isometopines exhibit sexual dimorphism regarding coloration and structure (Slater and Schuh 1969; Yasunaga et al. 2017), and the lack of a male description of *I. jejuensis* makes it challenging to thoroughly compare its identity with congeners. Therefore, on the identity of *I. jejuensis*, we restrict the morphological discussion to only females.

#### ﻿Morphology

Kerzhner (1988b) described the coloration of antennal segment II in females of *I. amurensis* as ‘whitish in female, with pale brown apex’; this condition is confirmed in the paralectotype female (Fig. 1I). On the other hand, Jung et al. (2015) mention that *I. amurensis* has ‘[uniformly] yellowish brown’ antennae. Kim and Jung (2016) described antennal segment II of *I. jejuensis* as ‘dark brown except for pale apex’. However, the pale apex in Kim and Jung (2016) actually refers to the extreme apex where segment III meets segment II; therefore, we regard the coloration of segment II of *I. jejuensis* as being uniformly dark brown.

Our re-examinations of female *I. amurensis* specimens from the Russian Far East and Korea (Figs 2A, B, 6C, D) revealed that the color pattern of the antennae can vary between pale brown with darker apex to uniformly brownish. Figs 2A, B, 6C, D show a series of specimens with varying antennal color collected from a single tree in Gwacheon-si, Korea, strongly suggesting that the color of the antennae is subject to variation among individuals. There are also reported cases in the genus *Isometopus* of which the color of the antennae is variable. The male *Isometopus hananoi* Hasegawa, 1946 illustrated in the figure of Tsunoda et al. (2020: fig. 6) shows two types of color pattern for antennal segment II: uniformly pale brown or pale brown with darker apex. Therefore, the color difference of antennal segment II cannot serve as a decisive character for unequivocally distinguishing *I. jejuensis* from *I. amurensis*.

Kim and Jung (2016) provided a schematic drawing of the sclerotized rings of *I. jejuensis* and argued that the structure of the apex of each sclerotized ring is different from *I. amurensis* (Kim and Jung 2016: fig. 4A, C). Dissection of selected *I. amurensis* females from South Korea showed that the structure of the sclerotized ring is variable even between specimens collected from close proximity (Fig. 3A–F). All dissected individuals have elongate-oval, teardrop-shaped thin-rimmed sclerotized rings (Fig. 3A, C, E). The sclerotized rings of the individual from Suwon (Fig. 3E) are similar to the shape illustrated for *I. jejuensis* by Kim and Jung (2016: fig. 4A), whereas the individual from Gwacheon (Fig. 3A) corresponds to the shape illustrated for *I. amurensis* in Jung et al. (2015: fig. 1D) and Kim and Jung (2016: fig. 4C). Considering that the dissected individuals were found in geographically close regions, the subtle variance observed in the sclerotized ring suggests that these structures cannot be used as a diagnostic character in distinguishing *I. jejuensis* from *I. amurensis*.

**Figure 4.**
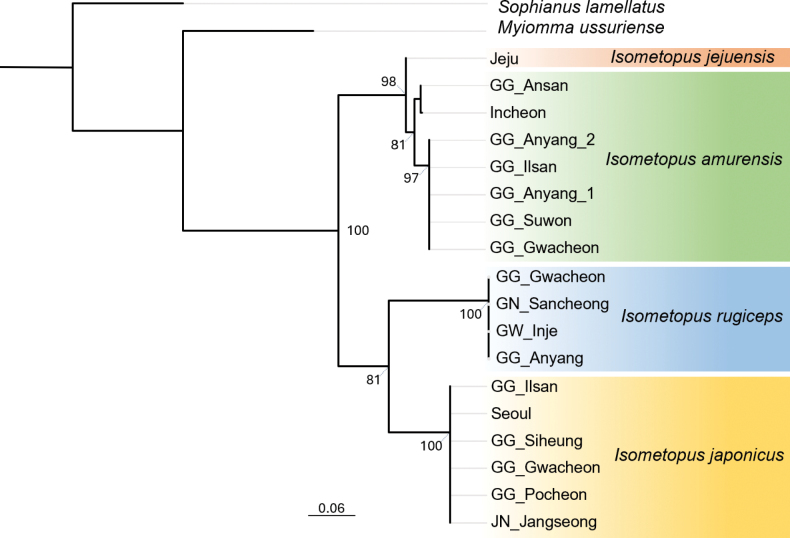
Maximum likelihood (ML) tree of selected *Isometopus* species based on COI sequences (511 bp).

Even though it was not mentioned as a diagnostic character differentiating *I. jejuensis* from *I. amurensis* by Kim and Jung (2016), the cuneal color pattern is different between the published descriptions of the two species. Kerzhner (1988b: 5, fig. 2) showed that the cuneus of *I. amurensis* is white, without any markings, which is confirmed with the actual condition found in the paralectotype (Fig. 1I). This coincides with the redescription of Jung et al. (2015: 599) of this species. In contrast, Kim and Jung (2016) described the cuneal coloration of *I. jejuensis* as being ‘anterior part of cuneus pale brown, apical part of cuneus grayish’. The cuneal coloration in *I. amurensis* is variable even between specimens collected from the same locality (Fig. 2C, D), and the cuneal color pattern of *I. jejuensis* (Kim and Jung 2016: 138, 143: figs 1A, 6A) corresponds to the color of *I. amurensis* in Fig. 2C.

Other diagnostic characters of *I. jejuensis* (e.g., coloration and punctation pattern of head, antennal proportion, vestiture of the dorsum, and general color pattern) commonly used in classifying *Isometopus* species (cf. Wagner and Weber 1964) are virtually identical to *I. amurensis*. Examination of the type specimens of *I. amurensis* and *I. jejuensis*, accompanied by the description and figure in Kim and Jung (2016: 138, 143: figs 1A, E, 6A) make it evident that it is conspecific with *I. amurensis*.

#### ﻿Molecular analysis

According to our maximum likelihood (ML) tree (Fig. 4), *I. jejuensis* sample (KY367033.1) from Jeju Island (33.43N, 126.62E) published by Kim and Jung (2018) is clearly nested within the clade of *I. amurensis* samples from various localities in South Korea, with a strong bootstrap support value (BS = 100).

Furthermore, the uncorrected pairwise sequence differences (*p*-distances) based on 511 bp mitochondrial COI sequences between *I. amurensis* and *I. jejuensis* were 1.76–2.73% (Table 2). Considering that in previous studies, the average intraspecific COI sequence distance for mirid species such as *Eurystylus coelestialium* (Kirkaldy, 1902) was reported as up to 2.8% (Kim and Jung 2018), we regard *I. jejuensis* (sample no. KY367037.1) genetically indistinct from other *I. amurensis* samples.

**Table 2.** Uncorrected pairwise distances for *Isometopus* species (*I. amurensis*, *I. japonicus*, *I. jejuensis*, and *I. rugiceps*), based on 511 bp mitochondrial COI sequences. Intraspecific *p*-distances are in bold font.

	Species	*n*	1	2	3	4
1	*Isometopus amurensis*	7	**0–2.54**			
2	*Isometopus jejuensis*	1	1.76–2.73	–		
3	*Isometopus japonicus*	6	13.30–14.09	13.11	**0.00**	
4	*Isometopus rugiceps*	4	14.67–15.06	14.67	11.74	**0.00**

#### ﻿Bionomics

Kerzhner (1988b) reported *I. amurensis* from the bark of *Quercus dentata* Thunb. and *Q. mongolica* Fisch. ex Ledeb. (Fagaceae) in the Russian Far East and Jung et al. (2015) reported a single female on the bark of *Taxus cuspidata* Siebold & Zucc. (Taxaceae) in South Korea. Kim and Jung (2016), in describing *I. jejuensis*, reported it from the bark of *Neolitsea sericea* (Blume) Koidz. (Lauraceae). This broadleaf is indigenous to warm temperate and subtropical East Asia, which is sparsely distributed in Jeju-do and Ulleng-do islands and the southwestern coastline in Korea (Lee and Choi 2010). These different host associations prompted Kim and Jung (2016) to argue the distinctiveness of *I. jejuensis* from *I. amurensis*.

However, many isometopine species are believed not to be host plant specific (Yasunaga and Hayashi 2002; Namyatova and Cassis 2016), and our field observation and literature review for species occurring in East Asia (Table 3) support this viewpoint. The nymphs of *Isometopus* species are found on the bark of trunks and branches of various broadleaved trees, and occasional adults are confirmed on plants that likely represent opportunistic habitats (e.g., *I. japonicus* on *Picea* sp., Yasunaga 2005). East Asian species have been reported on 3–10 different plant families, and some were even confirmed on non-native plants (i.e., *I. amurensis*, *I. hananoi*, and *I. japonicus*, see Table 3). Since many isometopine species are known to be predaceous and presumed to prey on small arthropods (Wheeler and Henry 1978; Yasunaga 2005; Namyatova and Cassis 2016; Tsunoda et al. 2020), the plant association is presumed not to play a significant role in the lifecycle of this group.

**Table 3.** Collection substrate of East Asian *Isometopus* species. ^!^ plant species with immatures confirmed; * plant species which are non-native within its distributional range, ^1^*F. griffithii* in Nagasaki, Kyushu is an introduced population possibly from the Ryukyus (Yasunaga et al. 2017).

Species	Collection substrate (plant bark)	Family	References	Locality
*Isometopus amurensis* Kerzhner, 1988	Betula pendula subsp. mandshurica (Regel) Ashburner & McAll. ^!*^	Betulaceae	This work	Korea
	*Albizia julibrissin* Durazz. ^!^	Fabaceae	This work	Korea
	*Styphnolobium japonicum* (L.) Schott ^!*^	Fabaceae	This work	Korea
	*Quercus dentata* Thunb.	Fagaceae	Kerzhner (1988b)	Russia (Far East)
	*Quercus mongolica* Fisch. ex Ledeb.	Fagaceae	Kerzhner (1988b)	Russia (Far East)
	*Quercus palustris* Münchh. ^!*^	Fagaceae	This work	Korea
	*Neolitsea sericea* (Blume) Koidz.	Lauraceae	Kim and Jung (2016)	Korea (Jeju)
	*Prunus × yedoensis* Matsum. ^!*^	Rosaceae	This work	Korea
	*Salix* sp. ^!^	Salicaceae	This work	Korea
	*Acer palmatum* Thunb. ^!^	Sapindaceae	This work	Korea
	*Styrax japonicus* Siebold & Zucc. ^!^	Styracaceae	This work	Korea (Jeju)
	*Taxus cuspidata* Siebold & Zucc.	Taxaceae	Jung et al. (2015)	Korea
	*Zelkova serrata* (Thunb.) Makino ^!^	Ulmaceae	This work	Korea
*Isometopus beijingensis* Ren & Yang, 1988	Unknown		Qi (2005)	China
*Isometopus bipunctatus* Lin, 2004	*Fraxinus griffithii* C.B.Clarke ^!^	Oleaceae	Yasunaga et al. (2017)	Taiwan
*Isometopus citri* Ren, 1987	*Prunus × yedoensis* Matsum. ^*^	Rosaceae	This work	Korea
	*Citrus maxima* (Burm.) Merr. ^*^	Rutaceae	Qi (2005)	China
	*Zelkova serrata* (Thunb.) Makino	Ulmaceae	This work	Korea
*Isometopus fasciatus* Hsiao, 1964	Unknown		Qi (2005)	China
*Isometopus hainanus* Hsiao, 1964	Unknown		Qi (2005)	China
*Isometopus hananoi* Hasegawa, 1946	*Quercus* sp.	Fagaceae	Yasunaga et al. (2017)	Japan
	*Fraxinus griffithii* C.B.Clarke ^*^	Oleaceae	Yasunaga et al. (2017)	Japan (Kyushu^1^)
	*Prunus* sp.	Rosaceae	Yasunaga et al. (2017)	Japan
	*Zelkova serrata* (Thunb.) Makino ^!^	Ulmaceae	Urayama et al. (2019) Tsunoda et al. (2020)	Japan
*Isometopus hasegawai* Miyamoto, 1965	*Pinus luchuensis* Mayr	Pinaceae	Yasunaga (2005)	Japan (Ryukyu)
*Isometopus ishigaki* Yasunaga, 2012	*Cinnamomum chekiangense* Nakai	Lauraceae	Yasunaga (2005)	Japan (Ryukyu)
	*Fraxinus griffithii* C.B.Clarke ^!^	Oleaceae	Yasunaga (2005)	Japan (Ryukyu)
*Isometopus japonicus* Hasegawa, 1946	Betula pendula subsp. mandshurica (Regel) Ashburner & McAll.	Betulaceae	Yasunaga (2001)	Japan
	*Robinia pseudoacacia* L. ^!*^	Fabaceae	This work	Korea
	*Castanopsis cuspidata* (Thunb.) Schottky	Fagaceae	Shishido et al. (2020)	Japan
	*Quercus palustris* Münchh. ^!*^	Fagaceae	This work	Korea
	*Picea* sp.	Pinaceae	Yasunaga (2005)	Japan
	*Prunus × yedoensis* Matsum. ^!*^	Rosaceae	This work	Korea
	*Prunus sargentii* Rehder	Rosaceae	Yasunaga (2001)	Japan
	*Sorbus commixta* Hedl.	Rosaceae	Yasunaga (2001)	Japan
	*Acer palmatum* Thunb. ^!^	Sapindaceae	This work	Korea
	*Taxus cuspidata* Siebold & Zucc.	Taxaceae	Yasunaga (2001)	Japan
	*Zelkova serrata* (Thunb.) Makino ^!^	Ulmaceae	Yasunaga (2001), Kim and Jung (2016), Urayama et al. (2019)	Japan, Korea
*Isometopus lini* Lin, 2004	Unknown		Yasunaga et al. (2017)	Taiwan
*Isometopus marginatus* Ren & Yang, 1988	Unknown		Qi (2005)	China
*Isometopus nigrosignatus* Ren, 1987	Unknown		Qi (2005)	China
*Isometopus puberus* Ren, 1991	Unknown		Qi (2005)	China
*Isometopus renae* Lin, 2004	Unknown		Yasunaga et al. (2017)	Taiwan
*Isometopus rugiceps* Kerzhner, 1988	*Albizia julibrissin* Durazz. ^!^	Fabaceae	This work	Korea
	*Robinia pseudoacacia* L. ^!*^	Fabaceae	This work	Korea
	*Quercus dentata* Thunb.	Fagaceae	Kerzhner (1988b)	Russia (Far East)
	Prunus (Cerasus) sp. ^!^	Rosaceae	This work	Korea
	*Zelkova serrata* (Thunb.) Makino ^!^	Ulmaceae	This work	Korea
*Isometopus shaowuensis* Ren, 1987	*Citrus* sp.	Rutaceae	Qi (2005)	China
*Isometopus takaii* Yasunaga, 2012	*Fraxinus griffithii* C.B.Clarke	Oleaceae	Yasunaga (2005)	Japan (Ryukyu)
*Isometopus tianjinus* Hsiao, 1964	*Styphnolobium japonicum* (L.) Schott	Fabaceae	Qi (2005)	China

Adults of *I. amurensis* were confirmed on the bark of 12 plant species in nine families (Table 3). Of these, immatures were confirmed from the bark of nine plant species in eight families. Among the confirmed breeding hosts, Betula pendula subsp. mandshurica (Regel) Ashburner & McAll. (Betulaceae) and *Quercus palustris* Münchh. (Fagaceae) are non-native plants in South Korea which have been imported for urban landscaping purpose (Korea National Arboretum 2010; Kim and Kim 2018). The wide and flexible habitat preference of *I. amurensis* shows that the different host plants cannot be used to distinguish the *N. sericea*-inhabiting population from that on Jeju Island.

#### ﻿Conclusion

**Figure 5.**
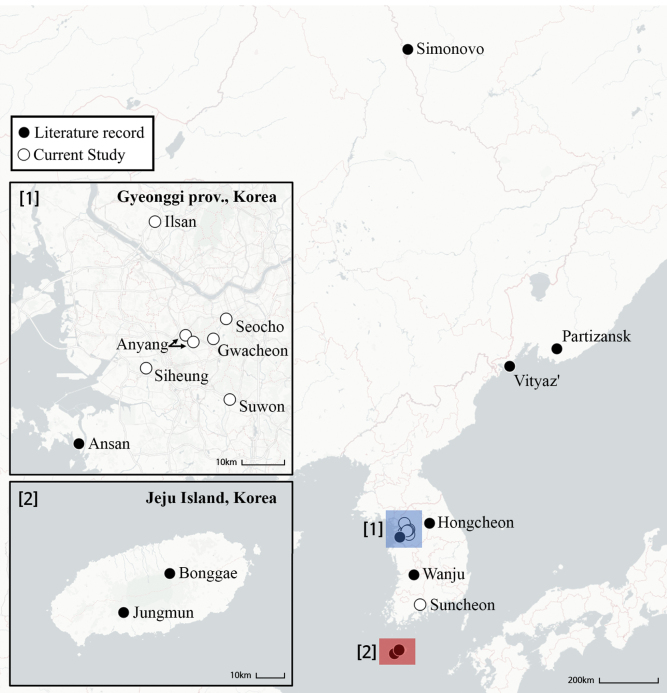
Distribution of *Isometopus amurensis* Kerzhner, 1988 in East Asia. Two localities in Jeju Island refer to records previously reported for *I. jejuensis* Kim & Jung, 2016

Based on the above evidence, we confirm that *Isometopus jejuensis* Kim & Jung, 2016 is a new subjective synonym of *Isometopus amurensis* Kerzhner, 1988. Accordingly, Korean Jeju Island is added to the distribution of *I. amurensis*, marking it the southernmost distribution record for the species (Fig. 5).

**Figure 6.**
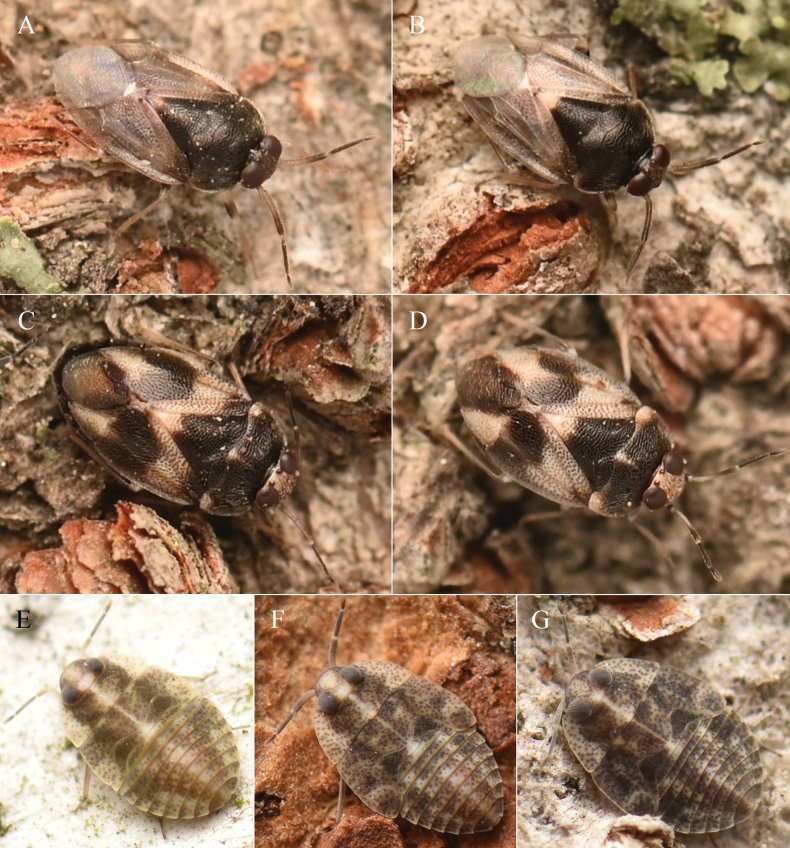
*Isometopus amurensis* Kerzhner, 1988, live specimens. A, B. Male; C, D. Female, E. Early-stage nymph; F, G. Final instar nymph.

#### ﻿Biology

In the western lowlands of South Korea (cf. Incheon, Siheung, Anyang, Gwacheon) adults appear in mid-June and mid-August, suggesting that this species has at least two generations per year. Immatures of various developmental stages were observed on the bark of *Acer palmatum* Thunb. (Sapindaceae), Betula pendula subsp. mandshurica (Regel) Ashburner & McAll. (Betulaceae), *Albizia julibrissin* Durazz. (Fabaceae), *Prunus × yedoensis* Matsum. (Rosaceae), *Salix* sp. (Salicaceae), *Styphnolobium japonicum* (L.) Schott (Fabaceae), *Styrax japonicus* Siebold & Zucc. (Styracaceae), *Quercus palustris* Münchh. (Fagaceae), and *Zelkova serrata* (Thunb.) Makino (Ulmaceae). Kerzhner (1988b) reported specimens collected at light from Primorsky Krai, Russia. Similarly, we collected a single male at an artificial light in Gwacheon, Korea.

Observed adults and nymphs actively moved along the bark surface and probed between the bark and mosses with their labium, likely searching for prey (Suppl. material 1). Multiple bark lice, small spiders, mites, and springtails were found on the bark along with *I. amurensis* and were assumed to be the possible prey items (Yasunaga 2005; Tsunoda et al. 2020). Other congeners, including *I. citri* Ren, 1987, *I. japonicus* Hasegawa, 1946, *I. rugiceps* Kerzhner, 1988 and *Myiomma ussuriense* Ostapenko, 2001 were often found on the same bark with *I. amurensis*. In a suburban park in Gwacheon-si, this species was found co-inhabiting the bark of *Z. serrata* with two hallodapine species, *Acrorrhinium inexpectatum* (Josifov, 1978) and *Cleotomiris josifovi* Konstantinov & Simov, 2014 (Phylinae: Hallodapini); these species possibly share and compete for prey.

### ﻿Notes on Korean species of the subfamily Isometopinae (Hemiptera: Miridae)

#### ﻿ Isometopus citri

Taxon classificationAnimaliaHemipteraMiridae

Ren, 1987

0E4141A1-8C94-5833-927D-870F58A26829

Fig. 7J–K

Isometopus citri Ren, 1987: 398. Holotype: (♀), China: Xixiang, Shaanxi; NKUM.
Isometopus citri : Zheng 1995: 460 (list), Kerzhner and Josifov 1999: 3 (catalogue, distribution), Schuh, 2002–2013 (web catalogue), Qi 2005: 511 (listed, distribution, host plant), Liu et al. 2022: 429 (redescription, distribution, host plant), Taszakowski et al. 2023: 185 (catalogue).

##### Material examined.

South Korea – **Gyeonggi-do.** • 2 ♀♀; Ilsan lake park, Goyang-si; 37°39.17'N, 126°45.85'E; 20.vi.2024; W. Kim leg. (SNU, UMMZ) – **Jeollanam-do.** • 1 ♀; 124, Byekgye-ro, Okryong-myeon, Gwangyang-si; 10.vi.2025; M. Oh leg. (SNU).

**Figure 7.**
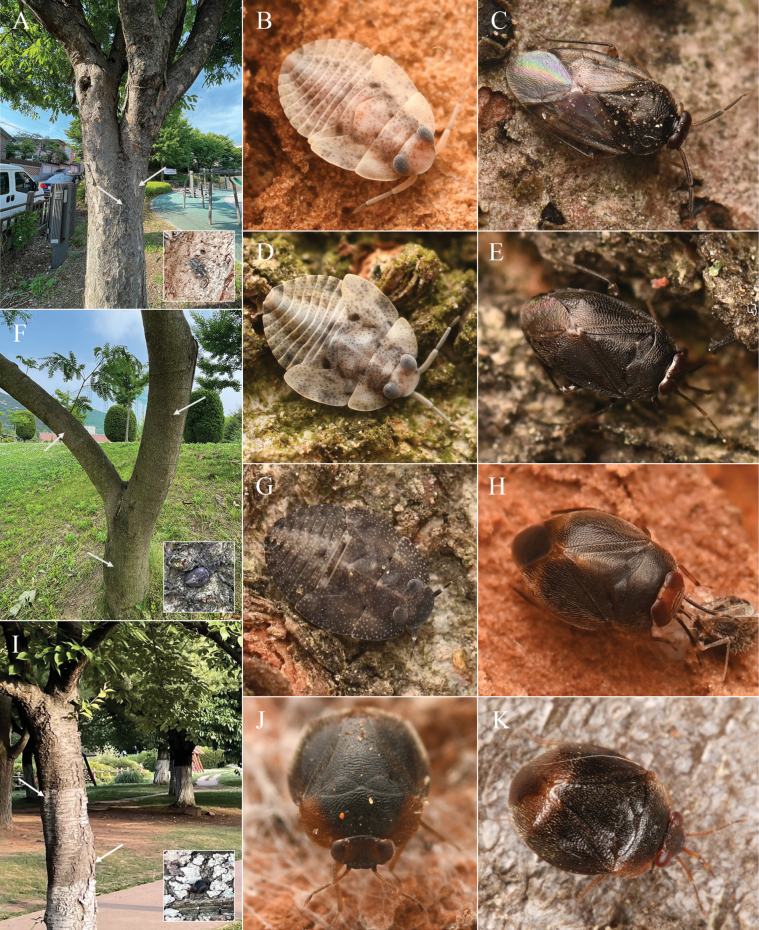
*Isometopus* species in South Korea. A–E. *Isometopus japonicus* Hasegawa, 1946. A. Habitat, on bark of *Zelkova serrata*; B. Early-stage nymph; C. Male; D. Final instar; E. Female; F–H. *I. rugiceps* Kerzhner, 1988. F. Habitat, on bark of *Albizia julibrissin*; G. Final instar; H. Female; I–K. *I. citri* Ren, 1987. I. Habitat, on bark of *Prunus × yedoensis*. J, K. Female.

##### Distribution.

China (Fujian, Shaanxi, Yunnan) (Liu et al. 2022), South Korea (Gyeonggi-do, Jeollanam-do).

##### Note.

This is a new record for Korea. Two females were collected from the bark of *Prunus × yedoensis* planted for ornamental purposes, where they were found with *I. amurensis* and *I. japonicus*. Another female from Gwangyang was collected from the bark of *Z. serrata*.

#### ﻿ Isometopus japonicus

Taxon classificationAnimaliaHemipteraMiridae

Hasegawa, 1946

6BFB35E9-3401-5DA3-8F9C-D1074AA303CF

Fig. 7A–E

Isometopus japonicus Hasegawa, 1946: 1. Holotype: (♂), Japan: Ōiso, Prov. Sagami, Honshu; NIAES.
Isometopus japonicus : Eyles 1971: 942 (list), Kerzhner and Josifov 1999: 4 (catalogue, distribution), Yasunaga 2001: 16, 118 (diagnosis, photo, host plant), Yasunaga and Hayashi 2002: 100 (checklist, distribution), Schuh 2002–2013 (web catalogue), Kim and Jung 2016: 140 (redescription), Ahn et al. 2018: 380 (diagnosis, photo); Urayama et al. 2019: 79 (distribution, photo), KSAE and ESK 2021: 155 (list), Taszakowski et al. 2023: 185 (catalogue).

##### Material examined.

South Korea – **Seoul.** • 2 nymphs; Umyeon-dong, Seocho-gu; 37°27.97'N, 127°01.19'E; 22.v.2024; W. Kim leg. (SCNU). – **Gyeonggi-do.** • 3 ♀♀; Jungang-dong, Gwacheon-si; 37°25.52'N, 126°59.27'E; 17.vi.2023; W. Kim leg. (SCNU) • 6 nymphs (4^th^ and 5^th^); same locality; 21.v.2024; W. Kim leg. (SCNU) • 1 nymph (5^th^); same locality and collector; 29.v.2024 (UMMZ) • 1 nymph (4^th^); Galhyeon-dong, Gwacheon-si; 37°25.25'N, 126°58.25'E; 21.v.2024; W. Kim leg. (UMMZ) • 5 ♂♂, 10 nymphs (4^th^ and 5^th^); Neunggok-dong, Siheung-si; 37°21.92'N, 126°48.83'E; 24.v.2024; W. Kim and J. Park leg. (SCNU) • 1 ♀; same locality; 28.vi.2024; W. Kim leg. (SCNU); • 1 ♀; Ilsan lake park, Goyang-si; 37°39.17'N, 126°45.85'E; 20.vi.2024; W. Kim leg. (SCNU).

##### Distribution.

Japan (Hokkaido, Honshu, Shikoku, Kyushu, Tsushima Island) (Yasunaga and Hayashi 2002), South Korea (Gyeonggi-do, Chungcheongbuk-do, Jeollabuk-do) (Ahn et al. 2018).

##### Note.

This species was described based on 40 specimens from eight localities in Honshu and Hokkaido in Japan, and was reported from various parts of temperate Japan until Kim and Jung (2016) reported it from South Korea based on seven males and five females, all from the single locality in Jeonju-si, Jeollabuk-do Province. The original description of Hasegawa (1946) mentioned the coloration of the hemelytra as ‘brownish ochraceous or blackish brown, base of the corium and embolium with a pale flavescent (or whitish) triangular patch…’ and subsequent studies including (Yasunaga 2001: pl. 2; fig. 5B, C) also depict similar characteristics. However, the Korean specimens reported by Kim and Jung (2016) does not have a conspicuous pale brown region on the base of corium (Kim and Jung 2016: 138, fig. 1C), which differs from their description of females that have the ‘hemelytra mostly dark brown; base of corium and embolium pale brown’ (Kim and Jung 2016: 142). Multiple specimens collected by the authors in various localities in Korea also have a uniformly dark brown hemelytra in females (Fig. 7E). Whether this color forms represent geographic variation could be resolved by future reexamination and comparative study of Korean and Japanese specimens.

We note that the live habitus photo provided by Ahn et al. (2018) has the male and female labels reversed. Kim and Jung (2016: 144) indicated that *Zelkova serrata* was a new host record for *I. japonicus*, but this is clearly an error since earlier Japanese literature mentioned this species from various deciduous trees, including *Z. serrata* (Yasunaga 2001: 118, also see Table 3).

In Korea, this species is commonly found but not restricted to the bark of *Z. serrata* (Table 3). Early stage nymphs (2^nd^–3^rd^) were observed in Pocheon-si, Gyeonggi-do (J. Park, pers. comm., April 2024) on 21 April 2024. The first author also observed a freshly emerged male on 24 May 2024 in Siheung-si. Based on the collection records, this species seems to have a univoltine lifecycle, where adults appear from late May to June in the central regions of the Korean Peninsula.

#### ﻿ Isometopus rugiceps

Taxon classificationAnimaliaHemipteraMiridae

Kerzhner, 1988

6B464E21-F4F7-5C33-9167-27C6F7DD24F5

Fig. 7F–H

Isometopus rugiceps Kerzhner, 1988a: 789. Lectotype (as holotype, see Kerzhner 1988b: 6): ♂, Russia: Vityaz’, 15 km south of Sukhanovka; ZISP.
Isometopus rugiceps : Kerzhner and Josifov 1999: 5 (catalogue, distribution), Schuh 2002–2013 (web catalogue), Ahn et al. 2018: 382 (diagnosis, photo), KSAE and ESK 2021: 155 (list), Taszakowski et al. 2023: 186 (catalogue).

##### Material examined.

South Korea – **Gyeonggi-do.** • 1 nymph (5^th^); Jungang-dong, Gwacheon-si; 37°25.54'N, 126°59.28'E; 21.v.2024; emerged as ♀ on 24.v.2024; W. Kim leg. (SCNU) • 1 ♀, 5 nymphs (5^th^); Manan-gu, Anyang-si; 37°25.96'N, 126°54.92'E; 24.v.2024; W. Kim and J. Park leg. (SCNU) • 1 ♂; same locality; 25.v.2024; J. Park leg. • 3 ♀♀; same locality; 30.v.2024; W. Kim leg. (SCNU). – **Gangwon-do.** • 1 ♀; Jindong-ri, Girin-myeon, Inje-gun; 576 m alt.; 13.vii.2016; Oh, Lee, Choi, Ahn leg. (SNU) • 4 ♀♀; Anheung-myeon, Hoengseong-gun; 37°27.79'N, 128°08.44'E; 18.vi.2023; W. Kim leg. (SCNU, UMMZ). – **Gyeongsangnam-do.** • 8 ♀♀; Sicheon-myeon, Sancheong-gun; 35°14.06'N, 127°47.37'E; 13.vi.2024; on light; W. Kim and S. Yang leg. (SCNU, UMMZ).

##### Distribution.

Russia (Far East: Primorsky Krai) (Kerzhner 1988b), South Korea (Gyeonggi-do, Gangwon-do, Gyeongsangnam-do, Jeollabuk-do) (Ahn et al. 2018).

##### Note.

This species is widely distributed in South Korea, where it inhabits the bark of multiple deciduous tree species (Table 3). Kerzhner (1988b) briefly mentioned three nymphs that might pertain to this species collected from Ryazanovka, Primorsky Krai, on the bark of *Quercus* sp. (Fagaceae) and *Ulmus* sp. (Ulmaceae), but no additional information is available. We present images of the final-instar nymphs for the first time (Fig. 6F); at first glance, it resembles *I. hananoi* Hasegawa, 1946 from temperate Japan in having a dark dorsum scattered with small, whitish spots (Tsunoda et al. 2020), but they can be distinguished by the spots being smaller and sparser, and the dark brown tergal segments with a medial bright region, its width approximately 1/4 of each abdominal segment.

Kerzhner (1988b) reported individuals collected at light in two localities from Primorsky Krai. Similarly, we collected eight females at a light trap in Sancheong-gun, Gyeongsangnam-do Province. Additionally, a single male was collected at light in Mt. Maisan, Jeollabuk-do Province (WG Kim, *pers. comm*.).

###### ﻿Tribe Myiommini Bergroth, 1924

**Genus *Myiomma* Puton, 1872**

#### ﻿ Myiomma ussuriense

Taxon classificationAnimaliaHemipteraMiridae

Ostapenko, 2001

D773483A-F03A-585C-8AAC-03625FEE2F8E

Fig. 8A–F

Myiomma ussuriensis : Ostapenko, 2001: 358. Holotype: ♀, Russia: 30 km NW of Arsen’ev, Primorsk Terr.; originally in Ostapenko’s collection, now deposited at ZISP.
Myiomma ussuriensis : Schuh, 2002–2013 (web catalogue), Taszakowski et al. (2023): 190 (catalogue).
Myiomma ussuriense : Kment and Carapezza 2017: 566 (list).
Myiomma koreana : Oh et al. 2025: 147. Holotype: ♂, Korea: Mt. Gaya, Seongju-gun; SNU. syn. nov.
Myiomma kukai : Jung and Lee 2012: 53 (misidentification), see Oh et al. 2025: 152.

##### Type material examined.

***Myiomma ussuriense* Ostapenko, 2001. *Holotype***: ♀, “[Primorsky krai, 30 km \ NW of Arsen’ev \ on *Fraxinus mandshurica* \ K. Ostapenko, 4.8.1999]” [handwritten] “*Holotypus* \ Myiomma \ ussuriensis \ Ostapenko det. 2000” [printed + handwritten] also with ZISP catalogue number: INS_HEM_0000126; deposited in ZISP. ***Myiomma koreanum* Oh & Lee, 2025. *Holotype***: ♂, “SNU \ Coll.#: 220806 MS-001 \ Loc.: Mt. Gaya, \ Seongju-gun, GB, Korea \ from light trap \ Date: 06.viii.2022 \ Leg.: WG. Kim” [printed] “HOLOTYPE \ *Myiomma koreana* \ Oh & Lee, 2025” [printed]; deposited in SNU.

**Figure 8.**
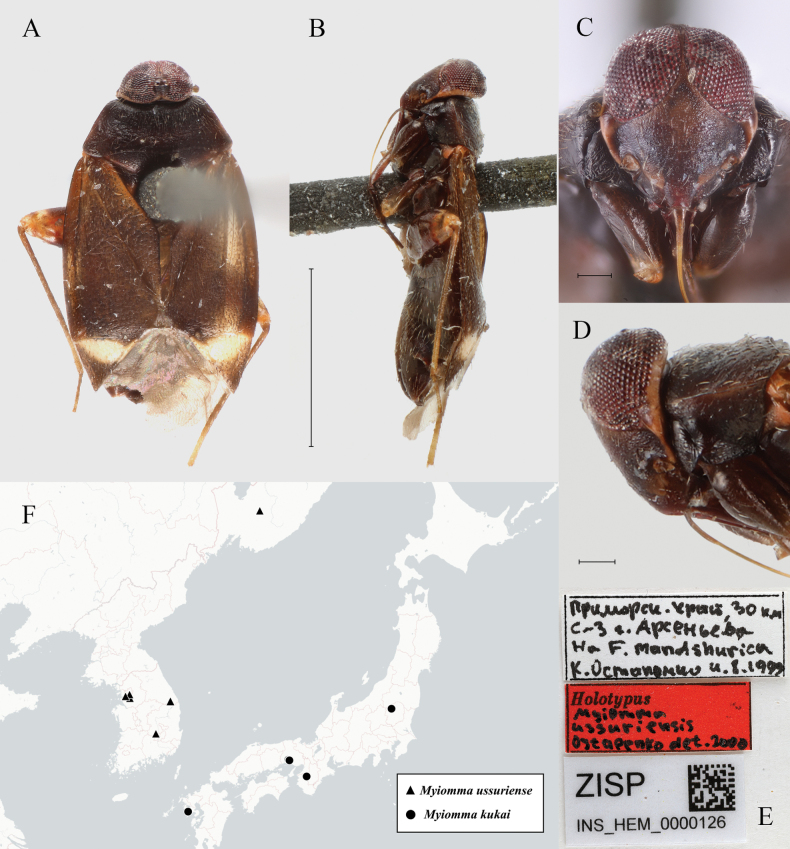
*Myiomma ussuriense* Ostapenko, 2001, holotype. A. Dorsal view; B. Lateral view; C. Head in frontal view; D. Head in lateral view; E. Labels; F. Distribution of *M. ussuriense* and *M. kukai* in East Asia. Scale bars: 1.0 mm (A, B); 0.1 mm (C, D). © ZISP (A–E).

##### Note.

Oh et al. (2025) described *M. koreana* based on eight specimens collected from four localities in South Korea, provided a description of both sexes, and briefly documented its bionomics. However, according to Kment and Carapezza (2017) the correct gender of *Myiomma* is neuter, and this species name should be amended as *M. koreanum* (ICZN 1999, article 31.2.). In describing *M. koreanum*, the authors were aware of *M. ussuriense*, which has been described based on a single female from Primorsky Krai, Russia, but have regarded the Korean specimens as distinct, mostly following Ostapenko’s (2001) original description of *M. ussuriense*.

Type specimens of *M. koreanum* collected from Korea showed differences with Ostapenko’s (2001) descriptions and figures, mainly following color and structures (original text of Ostapenko 2001: 358 in square brackets): 1) Structure of postocular rim viewed from the lateral side, 2) Inner margin of compound eye not conjoined [Eyes large, contacting along midline of head], 3) Blackish brown metafemur [hind femora pale brown, with diffuse subapical ring and apex yellow], 4) Dorsum densely covered with brown setae [covered dorsally with sparse recumbent hairs], 5) Clavus and corium uniformly dark brown [Inner margin of clavus and base of corium yellowish brown], 6) Metatibia widely dark brown, apical 1/4 pale [hind tibiae dark brown with yellowish apical half].

Photographic examination of the holotype of *M. ussuriense* housed at ZISP (Fig. 8A–E) showed that Ostapenko’s (2001) description is misleading, as it is different from the actual conditions found in the holotype. All morphological features of the female specimens of *M. koreanum* collected from Korea match well with the holotype of *M. ussuriense*, and we confirm that *Myiomma koreanum* Oh & Lee, 2025 is a new subjective synonym of *Myiomma ussuriense* Ostapenko, 2001.

Therefore, COI sequences previously submitted to GenBank, PQ530509, PQ530510 (Oh et al. 2025, as *M. koreana*) and GU194800 (Jung and Lee 2012, as *M. kukai*. See Oh et al. 2025: 151, 152) should be referred to as *M. ussuriense*. Kerzhner (1988b) mentioned a nymph of an undescribed *Myiomma* collected from Ryazanovka, Primorsky Krai, on the bark of *Quercus dentata*, but this species was never formally described, and could possibly pertain to *M. ussuriense*, based on the distribution of known *Myiomma* species from the Far East (Fig. 8F).

## Supplementary Material

XML Treatment for Isometopus amurensis

XML Treatment for Isometopus citri

XML Treatment for Isometopus japonicus

XML Treatment for Isometopus rugiceps

XML Treatment for Myiomma ussuriense

## ﻿Additional information

### Use of AI

No use of AI was reported.

### Author ORCIDs

Wonwoong Kim https://orcid.org/0000-0001-5837-1048

Minsuk Oh https://orcid.org/0000-0001-5185-0142

Geonho Cho https://orcid.org/0000-0003-1999-6963

### Data availability

All of the data that support the findings of this study are available in the main text or Supplementary Information.

## Supplementary materials

Supplementary material 1 Feeding attempts of *Isometopus* species in the natural habitat Data type mov Explanation note 1, *Isometopus japonicus*, early-stage nymph; 2, same, final instar nymph; 3, *I. amurensis*, final instar nymph; 4. *I. rugiceps*, adult female. https://binary.pensoft.net/file/1410131This dataset is made available under the Open Database License (http://opendatacommons.org/licenses/odbl/1.0/). The Open Database License (ODbL) is a license agreement intended to allow users to freely share, modify, and use this Dataset while maintaining this same freedom for others, provided that the original source and author(s) are credited.Wonwoong Kim

## References

[B1] Ahn SJ Kim WG Kim SS Park CG (2018) The terrestrial Heteroptera of Korea. Nature & Ecology, Seoul, 632 pp.

[B2] Akingbohungbe AE (1996) The Isometopinae (Heteroptera: Miridae) of Africa, Europe and the Middle East. Delar Tertiary Publisher, Ibadan, 174 pp.

[B3] Carvalho JCM (1951) New genera and species of Isometopidae in the collection of the British Museum of Natural History (Hemiptera). Anais da Academia Brasileira de Ciincias 23: 381–391.

[B4] Distant WL (1911) The Fauna of British India, Including Ceylon and Burma. Rhynchota. Vol. V. Heteroptera, appendix. Taylor & Francis, London, xii + 362 pp.

[B5] Edler D Klein J Antonelli A Silvestro D (2020) raxmlGUI 2.0: A graphical interface and toolkit for phylogenetic analyses using RAxML. Methods in Ecology and Evolution 12(2): 373–377. 10.1111/2041-210X.13512

[B6] Eyles AC (1971) List of Isometopidae (Heteroptera: Cimicoidea). New Zealand Journal of Science 14: 940–944.

[B7] Hasegawa H (1946) Descriptions of two new species of Isometopidae from Japan (Hemiptera, Heteroptera). Mushi 17: 1–4.

[B8] Hsiao TY (1964) New species and new record of Hemiptera-Heteroptera from China. Acta Zootaxonomica Sinica 1: 283–292.

[B9] ICZN [International Commission on Zoological Nomenclature] (1999) International Code of Zoological Nomenclature. 4th edn. The International Trust for Zoological Nomenclature, London, 306 pp. https://www.iczn.org/the-code/the-code-online/ [accessed 27 August 2024)

[B10] Josifov M (1978) Neue Miridenarten aus Nord-Korea (Heteroptera). Acta Entomologica Musei Nationalis Pragae. 39: 279–287.

[B11] Jung S Lee S (2012) Molecular phylogeny of the plant bugs (Heteroptera: Miridae) and the evolution of feeding habits. Cladistics 28: 50–79. 10.1111/j.1096-0031.2011.00365.x 34861758

[B12] Jung S Duwal RK Lee S (2015) A new record of the subfamily Isometopinae (Heteroptera: Miridae) from the Korean Peninsula. Zootaxa 3911(4): 598–600. 10.11646/zootaxa.3911.4.10 25661635

[B13] Katoh K Rozewicki J Yamada KD (2017) MAFFT online service: Multiple sequence alignment, interactive sequence choice and visualization. Briefings in Bioinformatics 20(4): 1160–1166. 10.1093/bib/bbx108 PMC6781576 28968734

[B14] Kerzhner IM (1988a) Infraorder Cimicomorpha. 21. Family Miridae (Capsidae). In: Ler PA (Ed.) Keys to the identification of insects of the Soviet Far East.Vol. 2, Nauka, Leningrad, 778–857. [in Russian]

[B15] Kerzhner IM (1988b) New and little known heteropterous insects (Heteroptera) from the Far East of the USSR. Vladivostok: Academy of Sciences, USSR, 1–84. [in Russian]

[B16] Kerzhner IM Josifov M (1999) Miridae Hahn, 1833. In: Aukema B, Rieger C (Eds) Catalogue of the Heteroptera of the Palaearctic Region, Vol. 3. The Netherlands Entomological Society, Amsterdam, 576 pp.

[B17] Kim J Jung S (2016) Taxonomic review of the genus *Isometopus* (Hemiptera: Miridae: Isometopinae) from the Korean Peninsula, with description of a new species. Zootaxa 4137(1): 137–145. 10.11646/zootaxa.4137.1.11 27395748

[B18] Kim J Jung S (2018) COI barcoding of plant bugs (Insecta: Hemiptera: Miridae). PeerJ 6: e6070. 10.7717/peerj.6070 PMC6284446 30533322

[B19] Kim T-Y Kim J-S (2018) Korean Trees. Dolbegae, Paju, 716 pp. [in Korean] 10.21215/kjfp.2018.8.5.716

[B20] Kirkaldy GW (1902) Memoir upon the Rhyncotal family Capsidae Auctt. Transactions of the Royal Entomological Society of London 50(2): 243–272. 10.1111/j.1365-2311.1902.tb01384.x

[B21] Kment P Carapezza A (2017) List of True bug taxa described by Rauno E. Linnavuori (Hemiptera: Heteroptera). Entomologica Americana 122(4): 528–621. 10.1664/1947-5144-122.4.528

[B22] Konstantinov F Neimorovets V (2025) (in press) Illustrated catalog of primary types of plant bugs (Hemiptera: Heteroptera: Miridae) in the Zoological Institute, Russian Academy of Sciences, St. Petersburg. European Journal of Taxonomy.

[B23] Konstantinov F Simov N (2014) A new *Cleotomiris* species (Hemiptera: Heteroptera: Miridae: Phylinae) from North Korea. Zootaxa 3786(1): 065–072. 10.11646/zootaxa.3786.1.4 24869523

[B24] Korea National Arboretum (2010) A Field Guide to Trees & Shrubs. Geobook, Seoul, 726 pp. [in Korean]

[B25] Korean Society of Applied Entomology [KSAE] & The Entomological Society of Korea [ESK] (2021) Check List of Insects from Korea. Paper and Pencil, Daegu, 1055 pp.

[B26] Kumar S Stecher G Li M Knyaz C Tamura K (2018) MEGA X: Molecular evolutionary genetics analysis across computing platforms. Molecular Biology and Evolution 35(6): 1547–1549. 10.1093/molbev/msy096 29722887 PMC5967553

[B27] Lee J-H Choi B-H (2010) Distribution and northernmost limit on the Korean Peninsula of three evergreen trees. Korean Journal of Plant Taxonomy 40(4): 267–273. 10.11110/kjpt.2010.40.4.267 [in Korean with English summary]

[B28] Lin C-S (2004) Seven new species of Isometopinae (Hemiptera: Miridae) from Taiwan. Formosan Entomologist 24: 317–326.

[B29] Lin C-S Yang C-T (2004) Isometopinae (Hemiptera: Miridae) from Taiwan. Formosan Entomologist 24: 27–42.

[B30] Liu G Mu Y Xu J Liu L (2022) Fauna Sinica. Insecta Vol. 73. HemipteraMiridae (III) Bryocorinae, Cylapinae, Deraeocorinae, Isometopinae and Psallopinae. Science Press, Beijing. 8 + 606pp. [xvii plates] [in Chinese with English abstract]

[B31] Namyatova AA Cassis G (2016) Review of the seven new species of Isometopinae (Heteroptera: Miridae) in Australia and discussion of distribution and host plant associations of the subfamily on a worldwide basis. Austral Entomology 55(4): 392–422. 10.1111/aen.12202

[B32] Oh M Lee S (2019) A review of the genus *Isometopus* (Hemiptera: Miridae: Isometopinae) in Korea. Journal of Asia-Pacific Biodiversity 12(4): 682–685. 10.1016/j.japb.2019.08.001

[B33] Oh M Kim S Lee S (2023) Revisiting the phylogeny of the family Miridae (Heteroptera: Cimicomorpha), with updated insights into its origin and life history evolution. Molecular Phylogenetics and Evolution 184: 107796. 10.1016/j.ympev.2023.107796 37086912

[B34] Oh M Kim W Kim WG Lee S (2025) A new species of the genus *Myiomma* (Heteroptera: Miridae: Isometopinae) from the Korean Peninsula. Zootaxa 5566(1): 145–158. 10.11646/zootaxa.5566.1.6 40173961

[B35] Ostapenko KA (2001) A new species of *Myiomma* from Primorsk Territory of Russia (Heteroptera: Miridae). Zoosystematica Rossica 9(2): 358.

[B36] POWO (2025) Plants of the World Online. Facilitated by the Royal Botanic Gardens, Kew. https://powo.science.kew.org/ [accessed 28 July 2025]

[B37] Qi B (2005) A taxonomic study of Isometopinae from China, including *Myiomma qinlingensis* sp. nov. (Hemiptera: Heteroptera: Miridae). Canadian Entomologist 137(5): 509–515. 10.4039/n04-114

[B38] Rambaut A (2018) FigTree v1.4.4. http://tree.bio.ed.ac.uk/software/figtree [accessed 10 Jan 2025]

[B39] Ren SZ (1987) New species and a newly recorded genus of Isometopidae from China. Acta Zootaxonomica Sinica 12: 398–403.

[B40] Ren SZ Yang CK (1988) New genus and new species of Isometopidae from China. Entomotaxonomia 10: 75–82.

[B41] Schuh RT (2002–2013) On-line Systematic Catalog of Plant Bugs (Insecta: Heteroptera: Miridae). http://research.amnh.org/pbi/catalog/ [accessed 9 September 2024]

[B42] Slater JA Schuh T (1969) New species of Isometopinae from South Africa (Hemiptera: Miridae). Journal of the Entomological Society of Southern Africa 32: 351–366.

[B43] Taszakowski A Kim J Bugaj-Nawrocka A Jung S (2023) Thirty years of progress in research on jumping tree bugs and the World checklist of Isometopinae (Hemiptera: Heteroptera: Miridae). Zootaxa 5382(1): 179–196. 10.11646/zootaxa.5382.1.19 38221265

[B44] Tsunoda R Takahama K Hinami H Tamada Y Yasunaga T Serrano Leon S Kawashita S Nagashima T (2020) Miscellaneous findings on the biology and morphology of Isometopus hasegawai (sic) (Miridae: Isometopinae). Rostria 65: 17–24. [in Japanese with English summary]

[B45] Urayama S Matsumoto K Yamamichi H Kawashita S Nagashima T Yasunaga T (2019) Heteropteran bugs found to inhabit *Zelkova* tree planted for landscaping at urbanized zones of Nagasaki City, Japan, close to ‘Hypocenter.’ Rostria 63: 77–84. [in Japanese with English summary]

[B46] Wagner E Weber HH (1964) Faune de France 67: Héteroptères Miridae. Fédération Française des Sociétés de Sciences Naturelles, Paris, 589 pp.

[B47] Wheeler AG Henry TJ (1978) Isometopinae (Hemiptera: Miridae) in Pennsylvania: Biology and descriptions of fifth instars, with observations of predation on obscure scale. Annals of the Entomological Society of America 71(4): 607–614. 10.1093/aesa/71.4.607

[B48] Yasunaga T (2001) Family Miridae Hahn, plant bugs. In: Yasunaga T, Takai M, Kawasawa T (Eds) A Field Guide to Japanese Bugs II. Terrestrial Heteropterans. Zenkoku Noson Kyoiku Kyokai Publ. Co. Ltd., Tokyo, 2–96 + 112–351. [in Japanese]

[B49] Yasunaga T (2005) Isometopine plant bugs (Heteroptera: Miridae), preferably inhabiting *Fraxinus griffithii*, on Ishigaki Island of the Ryukyus, Japan. Tijdschrift voor Entomologie 148(2): 341–349. 10.1163/22119434-900000179

[B50] Yasunaga T Hayashi M (2002) New or little known Isometopine plant bugs from Japan (Heteroptera: Miridae: Isometopinae). Tijdschrift voor Entomologie 145(1): 95–101. 10.1163/22119434-900000103

[B51] Yasunaga T Schwartz MD Aukema B (2012) Availability and type depository of twelve Japanese plant bug species (Hemiptera: Heteroptera: Miridae). Zootaxa 3478(1): 111–112. 10.11646/zootaxa.3478.1.13

[B52] Yasunaga T Yamada K Tsai J-F (2017) Taxonomic review of the plant bug subfamily Isometopinae for Taiwan and Japanese Southwest Islands, with descriptions of new taxa (Hemiptera: Heteroptera: Miridae: Isometopinae). Zootaxa 4365(4): 421–439. 10.11646/zootaxa.4365.4.3 29686197

[B53] Yeshwanth HM Chérot F Henry TJ (2021) The Isometopinae (Hemiptera: Heteroptera: Miridae) of India and Sri Lanka: A Review of the Subfamily, with Descriptions of Six New Species. Zootaxa 4903(2): 151–193. 10.11646/zootaxa.4903.2.1 33757094

